# Cone-Beam-CT Guided Adaptive Radiotherapy for Locally Advanced Non-small Cell Lung Cancer Enables Quality Assurance and Superior Sparing of Healthy Lung

**DOI:** 10.3389/fonc.2020.564857

**Published:** 2020-12-09

**Authors:** Philipp Hoegen, Clemens Lang, Sati Akbaba, Peter Häring, Mona Splinter, Annette Miltner, Marion Bachmann, Christiane Stahl-Arnsberger, Thomas Brechter, Rami A. El Shafie, Fabian Weykamp, Laila König, Jürgen Debus, Juliane Hörner-Rieber

**Affiliations:** ^1^Department of Radiation Oncology, Heidelberg University Hospital, Heidelberg, Germany; ^2^Heidelberg Institute of Radiation Oncology (HIRO), Heidelberg, Germany; ^3^National Center for Tumor Diseases (NCT), Heidelberg, Germany; ^4^Clinical Cooperation Unit Radiation Oncology, German Cancer Research Center (DKFZ), Heidelberg, Germany; ^5^Medical Physics in Radiotherapy, German Cancer Research Center (DKFZ), Heidelberg, Germany; ^6^Department of Radiation Oncology, Mainz University Hospital, Mainz, Germany; ^7^Heidelberg Ion-Beam Therapy Center (HIT), Department of Radiation Oncology, Heidelberg University Hospital, Heidelberg, Germany; ^8^German Cancer Consortium (DKTK), Heidelberg, Germany

**Keywords:** lung cancer, non-small cell lung cancer, adaptive radiotherapy, cone-beam computed tomography, organs at risk, quality assessment, pneumonitis, normal tissue

## Abstract

**Purpose:**

To evaluate the potential of cone-beam-CT (CB-CT) guided adaptive radiotherapy (ART) for locally advanced non-small cell lung cancer (NSCLC) for sparing of surrounding organs-at-risk (OAR).

**Materials and Methods:**

In 10 patients with locally advanced NSCLC, daily CB-CT imaging was acquired during radio- (n = 4) or radiochemotherapy (n = 6) for simulation of ART. Patients were treated with conventionally fractionated intensity-modulated radiotherapy (IMRT) with total doses of 60–66 Gy (pPlan) (311 fraction CB-CTs). OAR were segmented on every daily CB-CT and the tumor volumes were modified weekly depending on tumor changes. Doses actually delivered were recalculated on daily images (dPlan), and voxel-wise dose accumulation was performed using a deformable registration algorithm. For simulation of ART, treatment plans were adapted using the new contours and re-optimized weekly (aPlan).

**Results:**

CB-CT showed continuous tumor regression of 1.1 ± 0.4% per day, leading to a residual gross tumor volume (GTV) of 65.3 ± 13.4% after 6 weeks of radiotherapy (p = 0.005). Corresponding PTVs decreased to 83.7 ± 7.8% (p = 0.005). In the actually delivered plans (dPlan), both conformity (p = 0.005) and homogeneity (p = 0.059) indices were impaired compared to the initial plans (pPlan). This resulted in higher actual lung doses than planned: V_20Gy_ was 34.6 ± 6.8% instead of 32.8 ± 4.9% (p = 0.066), mean lung dose was 19.0 ± 3.1 Gy instead of 17.9 ± 2.5 Gy (p = 0.013). The generalized equivalent uniform dose (gEUD) of the lung was 18.9 ± 3.1 Gy instead of 17.8 ± 2.5 Gy (p = 0.013), leading to an increased lung normal tissue complication probability (NTCP) of 15.2 ± 13.9% instead of 9.6 ± 7.3% (p = 0.017). Weekly plan adaptation enabled decreased lung V_20Gy_ of 31.6 ± 6.2% (−3.0%, p = 0.007), decreased mean lung dose of 17.7 ± 2.9 Gy (−1.3 Gy, p = 0.005), and decreased lung gEUD of 17.6 ± 2.9 Gy (−1.3 Gy, p = 0.005). Thus, resulting lung NTCP was reduced to 10.0 ± 9.5% (−5.2%, p = 0.005). Target volume coverage represented by conformity and homogeneity indices could be improved by weekly plan adaptation (CI: p = 0.007, HI: p = 0.114) and reached levels of the initial plan (CI: p = 0.721, HI: p = 0.333).

**Conclusion:**

IGRT with CB-CT detects continuous GTV and PTV changes. CB-CT-guided ART for locally advanced NSCLC is feasible and enables superior sparing of healthy lung at high levels of plan conformity.

## Introduction

Lung cancer, with an estimated 388,000 deaths (one-fifth of the total) in 2018, remains the leading cause of cancer mortality in Europe (1). Surgical resection is the treatment of choice for non-small cell lung cancer (NSCLC), however, many patients are classified medically or technically inoperable due to locoregional tumor extension or severe comorbidities (2). For inoperable patients affected by locally advanced NSCLC, radiotherapy with a total dose of 60–70 Gy, if possible in combination with chemotherapy, represents the primary treatment modality (3, 4).

Despite substantial technological innovations during the last decades, damage to normal lung and heart tissue remains a major concern for patients’ morbidity and survival following radiochemotherapy (5–7). Indeed, symptomatic radiation pneumonitis is detected in up to 10–20% of locally advanced NSCLC patients undergoing curatively intended radiotherapy (8, 9). Development of radiation pneumonitis is further known to be an independent negative prognostic factor for survival (10). A recent study even underlined the predictive impact of lung dose on survival analyzing prognostic factors in 468 patients with stage IIIA-IIIB NSCLC (6). Regarding heart injury, a significant association between cardiac radiation doses and electrocardiographic (ECG) changes has been described (11). Furthermore, a recent study yet reported heart dose to be an independent dosimetric predictor of overall survival for locally advanced NSCLC patients (7).

Tumor shrinkage is frequently observed during radiochemotherapy of locally advanced NSCLC (12–14). Guckenberger et al. detected a continuous tumor regression by 1.2% per day during simultaneous radiochemotherapy (15). Therefore, frequent adaptation of the target volume and hence the treatment plan might be applied to limit toxicity by reducing dose to adjacent critical structures. In previous studies, however, adaptation was usually performed only once or twice during the course of radiotherapy (15–19).

In addition, many prior studies applied additional diagnostic computed tomography (CT) or positron emission tomography with computed tomography (PET/CT) for re-planning (18, 20–28). The regular application of diagnostic CT, if necessary with additional intravenous contrast agent, is personnel and time-consuming as well as associated with a not negligible additional radiation dose exposure to the patient (29). However, in modern radiotherapy daily image guidance is routinely performed with low-dose CT (e.g. cone-beam CT (CB-CT)) to account for position inaccuracies.

Thus, the aim of the current planning study is to evaluate the potential of continuous cone-beam CT-guided adaptive radiotherapy for sparing dose to surrounding organs-at-risks and eventually toxicity.

## Materials and Methods

The current study is based on 10 randomly selected patients with histologically proven stage III NSCLC who were treated between 09/2018 and 05/2019 at Heidelberg University Hospital, Germany. Treatment was decided upon by an interdisciplinary tumor board. Patients were classified to be technically or medically inoperable and were therefore treated with definitive radiochemotherapy (n = 6) or radiotherapy (n = 4). Due to a reduced performance status and severe comorbidities, chemotherapy could not be administered in four patients. Patient and treatment characteristics are illustrated in **Table 1**. Mutation analyses were only performed in four patients, however neither an EGFR mutation nor an EML4-ALK translocation was detected. PD-L1 expression ≥1% [in median 10% (1–90%)] was found in all patients (n = 7) for whom PD-L1 testing was performed. The analysis was approved by the Ethics Committee of the University Hospital Heidelberg (S-278/2019).

**Table 1:** Patient and treatment characteristics.

Patient	Age [years]	Sex	Karnofsky performancescore [%]	Clinical T N stage	UICC stage	Histology	GTV [cm^3^]	Radiotherapy dose (TD, SD) [Gy]	Chemotherapy	Agent
***1***	57.5	m	80	T4N3	IIIC	SCC	261.7	66, 2	yes	carboplatin/vinorelbine
***2***	62.6	m	80	T3N1	IIIA	Adeno-CA	185.2	64, 2	yes	carboplatin/pemetrexed
***3***	69.7	m	70	T4N3	IIIC	SCC	186.8	66, 2	yes	carboplatin/vinorelbine
***4***	81.6	f	80	T4N2	IIIB	SCC	99.7	60, 2	yes	carboplatin/vinorelbine
***5***	74.7	m	80	T4N0	IIIA	SCC	179.0	66, 2	no	
***6***	63.6	m	80	T4N1	IIIA	Adeno-CA	154.4	60, 2	yes	carboplatin/vinorelbine
***7***	79.0	m	60	T4N1	IIIA	Adeno-Ca	496.8	66, 2	no	
***8***	54.1	f	80	T4N3	IIIC	Adeno-Ca	383.1	60, 2	no	
***9***	57.7	f	90	T4N3	IIIC	Adeno-Ca	124.0	60, 2	yes	carboplatin/vinorelbine
***10***	86.5	f	70	T4N1	IIIA	SCC	220.5	66, 2	no	

F, female; m, male; Adeno-CA, adenocarcinoma; SCC, squamous cell carcinoma; GTV, gross tumor volume in planning CT; TD, total dose; SD, single dose; Gy, Gray.

### Treatment Planning and Dose Accumulation

For radiotherapy planning, native and contrast-enhanced CT scans with a slice thickness of 3 mm were acquired. The macroscopic primary tumor as well as the mediastinal and/or supraclavicular lymph node metastases were delineated as the gross tumor volume (GTV). The clinical target volume (CTV) comprised the GTV enlarged by a 6 mm margin as well as all involved lymph node regions. Additionally, the ipsilateral lymph node regions 4, 10, and 7 were always included into the CTV. The planning target volume (PTV) was generated by adding a 6 mm margin to the CTV. Patients were treated with volumetric arc therapy (VMAT) using 6 MV photons at the Elekta Versa HD (Elekta Instrument AB, Stockholm, Sweden). Radiation was delivered in 2.0 Gy fractions daily for 5 days per week to a total dose of 60.0–66.0 Gy. A mean dose of 60.0–66.0 Gy was prescribed to the PTV with a D95 (minimum dose of 95% of the reference volume) of 95% of the prescribed dose [primary plan (pPlan)]. Normal tissue dose volume constraints were in accordance with the current version of the NCCN guideline (version 3.2020) (30). In detail, the maximum dose to the spinal cord was limited to 45 Gy. A mean lung dose (MLD) ≤20 Gy as well as the volume of the whole lung (excluding the GTV) exceeding 20 Gy ≤ 35% were intended. Regarding the heart, the mean dose had to be ≤20 Gy with a volume exceeding 50 Gy ≤ 25%. For the esophagus, a mean dose ≤34 Gy was tolerated.

Daily cone-beam CTs were performed for image-guidance during treatment. Careful attention was paid to depict the whole target volume area including both lungs. Areas outside the CB-CT field of view were reconstructed using the outer body contour from the planning CT with density set to water. The target volumes were depicted completely on every CB-CT, so adjustment for missing target volume representation on CB-CTs was not needed. Images were imported into the Raystation treatment planning system, version 8.0 (RaySearch Laboratories AB, Stockholm, Sweden) and registered rigidly with the help of the performed treatment position alignments. Organs-at-risk (OAR) were delineated on every daily CB-CT. GTV and consequently CTV and PTV were adapted weekly depending on tumor changes. The delineated structures were used to support a deformable image registration between each CB-CT and the planning CT. For deformable image registration, Raystation’s built-in and validated algorithm ANACONDA was used (31–33).

In a first step, the initial treatment plan (pPlan) was calculated on the aligned CB-CT images to simulate the dose actually delivered [actual delivered plan (dPlan)]. In a second step, for simulation of adaptive radiotherapy (ART), treatment plans were adapted using the new contours and re-optimized weekly for every fifth fraction [adapted plan (aPlan)]. In detail, the initial treatment plan was applied for the 1^st^ fraction. The first plan adaptation was performed based on the first CB-CT and was applied for the following 4 days of treatment (fractions 2–5), while the second adapted plan (5^th^ CB-CT) was used for the following 5 days of treatment (fractions 6–10). The third adapted plan (10^th^ CB-CT) was again applied for the next 5 days of treatment (fractions 11–15), and so on. Identical planning objectives regarding PTV coverage and dose homogeneity as well as normal dose tissue volume constraints were used for the adapted plans.

Dose accumulation was done in a two-step process. First the vector field of the deformable image registration was used to project each CB-CT dose distribution onto the planning CT or CB-CT used for the previous plan adaptation. On the respective CT or CB-CT, these projected dose distributions were summed up to an accumulated dose distribution in a second step. Adapted plans were generated on the current CB-CT by using the corresponding (i.e. until then) accumulated dose as background dose. This background dose was incorporated in the optimization process of the adapted plan.

CB-CTs are generally prone to variable correlations between tissue density and respective Hounsfield units. For dose calculation, this was taken into account by using Raystation’s built-in and validated RS_auto_ approach (34).

For evaluation purposes, all CB-CT dose distributions were projected onto the planning CT to generate accumulated dose distributions. The accumulated dose distributions for pPlan, dPlan, and aPlan were analyzed with respect to differences in doses to the GTV, CTV, PTV, lungs, heart, esophagus, and spinal cord. Parameters of 3D dose distribution include mean dose, doses at X% volume (D_X%_), and volume percentage at doses of X Gy (V_XGy_). Maximum doses were assessed as near maximum doses D_2%_ according to ICRU 83 (35). The applied conformity index (CI) is defined as

$$CI=\frac{{\left(PTV\left(PIV\right)\right)}^{2}}{PTV*PIV}$$

with the prescribed isodose volume (PIV) and PTV(PIV) being the PTV covered by the PIV (36).

Homogeneity index (HI) is defined as follows (37):

$$HI=\frac{D5\%}{D95\%}$$

The equivalent uniform dose (EUD), generalized equivalent uniform dose (gEUD), normal tissue complication probability (NTCP), and tumor control probability (TCP) according to Niemierko et al (38, 39). are defined as:

$$EUD={\left(\sum_{i=1}\left(v_{i}D_{i}^{a}\right)\;\right)}^{\frac{1}{a}}$$

$$gEUD={\left(\frac{1}{N}\sum_{i=1}^{N}D_{i}^{a}\right)}^{\frac{1}{a}}$$

$$NTCP=\frac{1}{1+{\left(\frac{TD_{50}}{EUD}\right)}^{4y50}}$$

with TD_50_ as the tolerance dose for a 50% complication rate,

$$TCP=\frac{1}{1+{\left(\frac{TCD_{50}}{EUD}\right)}^{4y50}}$$

with TCD_50_ as the tumor dose to control 50% of the tumor.

Values for a, y50, TD_50_, and TCD_50_ were set based on publications by Okunieff et al (40). and Gay and Niemierko (38). For the spinal cord, parameters were lacking in the published literature and were therefore estimated in analogy to the optic nerve. Specifically, a was set to 3 (heart), 25 (spinal cord), 19 (esophagus), 1 (lung), −10 (GTV and PTV). Y50 was 3 (heart), 3 (spinal cord), 4 (esophagus), 2 (lung), 1.81 (GTV, PTV). TD_50_ was 50 (heart), 65 (spinal cord), 68 (esophagus), 24.5 (lung). TCD_50_ was 51.97 (GTV, PTV).

As the EUD value is very sensitive to single voxel values (41), a near maximum (D_1%_) and near minimum (D_99%_) approach was used in analogy to ICRU recommendations to reduce single voxel sensitivity.

### Statistical Analysis

Statistical analysis was performed with SPSS version 25.0 (IBM Corporation, Armonk, NY, USA) applying the nonparametric Wilcoxon signed-rank test for pairwise comparison of dependent, continuous, not normally distributed data. Significance was noted for p-values of <0.050.

## Results

### Tumor Regression During Radio(chemo)therapy

The mean GTV size in the planning CT was 229.1 cm³ (range 99.7–496.8 cm³). Continuous tumor shrinkage was detected during treatment (see **Table 2**), which corresponds to a mean regression of 1.1 ± 0.4% per fraction. This resulted in a residual gross tumor volume (GTV) of 65.3% (range 46.9–94.5%) after 6 weeks of radiotherapy (p = 0.005). At the end of treatment, mean GTV was 146.7 cm³ (range 72.4–326.5 cm³). Highest GTV shrinkage was observed in week 4 (mean shrinkage −11.9%), followed by week 3 (−9.6%).

**Table 2:** Size of gross (GTV) and planning target volumes (PTV) over the course of radiotherapy (negative values correspond to shrinkage).

	Planning – 1^st^ fraction	Week 1	Week 2	Week 3	Week 4	Week 5	Week 6	Week 7
Mean GTV shrinkage [%]	+2.9	-1.6	-6.7	-9.6	-11.9	-7.4	-5.1	-3.4
Mean PTV shrinkage [%]	+2.7	-2.4	-3.1	-3.3	-3.3	-3.1	-4.7	-3.2

The median interval between acquisition of the planning CT and start of radiotherapy was 7.5 days (range: 2–12 days). Five patients had GTV progression (range: 6.6–12.1%) from planning to first fraction. For these patients, median interval from planning to start of radiotherapy was 9 days (range: 7–12 days).

**Figure 1:**
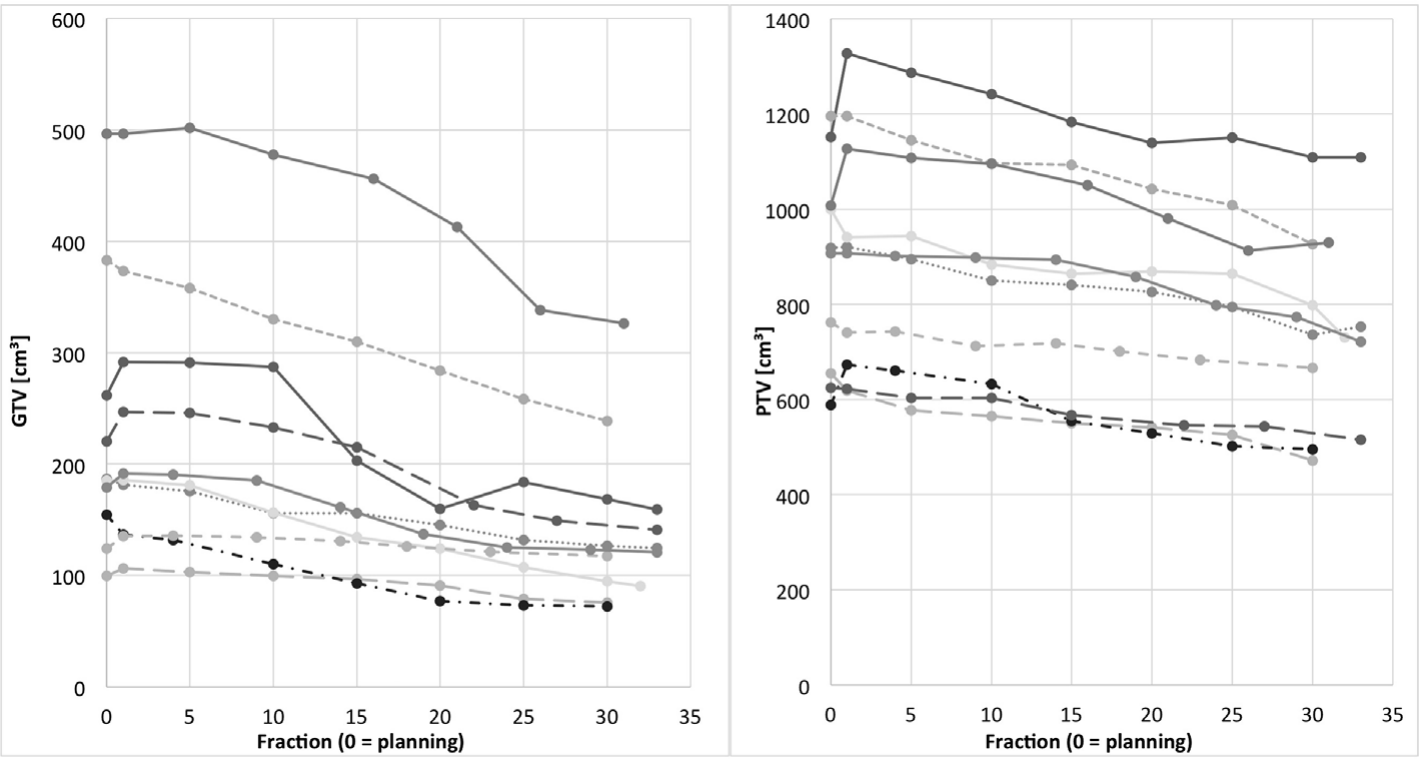
Individual GTV (left) and PTV (right) over the course of radiotherapy for all patients (fraction 0 = planning). GTV and PTV of the same individual patients are represented by the same line type.

Mean PTV size during planning was 881.1 cm³ (range 589.2–1,195.8 cm³). At the end of treatment, mean size decreased to 732.1 cm³ (range 472.0–1,109.0 cm³). Relative residual PTV was 83.7% (range: 72.1–96.3%) (p = 0.005). **Figure 1** shows absolute GTV and PTV sizes for all patients over the course of radio(chemo)therapy.

### Comparison Between Planned Doses, Accumulated Delivered Doses, and Dosimetric Effects of Adaptation

In comparison to the initial plans (pPlan), both conformity (p = 0.005) and homogeneity (p = 0.059) indices were reduced in the actually delivered plans (dPlan), see **Table 3**. Weekly plan adaptation allowed to restore target volume coverage represented by conformity and homogeneity indices (aPlan compared to dPlan: CI: p = 0.007, HI: p = 0.114) to similar levels as achieved in the initial plans (aPlan compared to pPlan: CI: p = 0.721, HI: p = 0.333).

**Table 3:** Conformity and homogeneity indices in the primary plan (pPlan), actually delivered plan (dPlan) and adapted plan (aPlan). All values given as mean ± standard deviation.

	Conformity index	Homogeneity index
**pPlan**	0.87 ± 0.04	1.09 ± 0.03
**dPlan**	0.79 ± 0.08	1.11 ± 0.04
**aPlan**	0.86 ± 0.05	1.09 ± 0.03

Dosimetric parameters of OAR are illustrated in **Table 4**. The generalized equivalent uniform dose (gEUD), normal tissue complication probability (NTCP), and tumor control probability (TCP) for different scenarios are summarized in **Table 5**.

**Table 4:** Organ at risk doses in the primary plan (pPlan), actually delivered plan (dPlan) and adapted plan (aPlan). All values given as mean ± standard deviation.

	Mean lung dose [Gy]	Lung V_20Gy_ [%]	Mean esophagus dose [Gy]	Esophagus D max [Gy]	Mean heart dose [Gy]	Spinal cord D max [Gy]
**pPlan**	17.9 ± 2.5	32.8 ± 4.9	28.5 ± 5.2	62.5 ± 2.9	9.7 ± 6.3	32.4 ± 5.2
**dPlan**	19.0 ± 3.1	34.6 ± 6.8	29.5 ± 5.9	61.6 ± 2.3	11.9 ± 5.9	32.0 ± 5.6
**aPlan**	17.7 ± 2.9	31.6 ± 6.2	28.7 ± 5.7	62.5 ± 2.7	11.2 ± 6.8	31.6 ± 5.3

Gy, Gray; D max, maximum dose.

**Table 5:** Generalized equivalent uniform dose (gEUD), normal tissue complication probability (NTCP) and tumor control probability (TCP) in organs at risk and planning target volume (PTV) for primary plan (pPlan), actually delivered plan (dPlan) and adapted plan (aPlan). All values given as mean ± standard deviation.

	gEUD Lung [Gy]	NTCP Lung [%]	gEUD Esophagus [Gy]	NTCP Esophagus [%]	gEUD Heart [Gy]	NTCP Heart [%]	gEUD Spinal Cord [Gy]	NTCP Spinal Cord [%]	gEUD PTV [Gy]	TCP PTV [%]
**pPlan**	17.8 ± 2.5	9.6 ± 7.3	56.0 ± 2.7	5.2 ± 3.5	16.0 ± 10.0	0.1 ± 0.2	29.3 ± 5.0	0.0 ± 0.1	62.6 ± 2.8	78.8 ± 5.6
**dPlan**	18.9 ± 3.1	15.2 ± 13.9	55.5 ± 2.5	4.5 ± 3.3	18.9 ± 10.0	0.1 ± 0.1	28.9 ± 5.4	0.0 ± 0.1	62.6 ± 2.6	78.0 ± 5.2
**aPlan**	17.6 ± 2.9	10.0 ± 9.5	56.0 ± 2.4	5.0 ± 3.4	18.1 ± 10.4	0.1 ± 0.4	28.6 ± 5.0	0.0 ± 0.1	62.5 ± 3.1	78.5 ± 6.1

Gy, Gray.

Actually applied cumulated dose to the lung was markedly higher than planned: V_20Gy_ was 34.6 ± 6.8% instead of 32.8 ± 4.9% (p = 0.066), mean lung dose was 19.0 ± 3.1 Gy instead of 17.9 ± 2.5 Gy (p = 0.013). The gEUD of the lung was 18.9 ± 3.1 Gy instead of 17.8 ± 2.5 Gy (p = 0.013), leading to an increased lung normal tissue complication probability (NTCP) of 15.2 ± 13.9% instead of 9.6 ± 7.3% (p = 0.017).

Weekly plan adaptation allowed for decreased lung V_20Gy_ of 31.6 ± 6.2% (−3.0%, p = 0.007), mean lung dose of 17.7 ± 2.9 Gy (−1.3 Gy, p = 0.005), and lung gEUD of 17.6 ± 2.9 Gy (−1.3 Gy, p = 0.005). Thus, resulting lung NTCP was reduced to 10.0 ± 9.5% (−5.2%, p = 0.005).

Regarding esophagus, heart, and spinal cord, mean and maximum doses as well as gEUD and NTCP did not differ significantly between planned, applied, and adapted plans (p > 0.050, respectively). Furthermore, no significant differences were detected for PTV gEUD, and tumor control probability between the three different scenarios (p > 0.050, respectively).

## Discussion

To the best of our knowledge, the present work is the first study using CB-CT for unconditional (i.e., not only if certain criteria are fulfilled) weekly adaptive radiotherapy in NSCLC. CB-CT showed continuous GTV regression during radiotherapy of locally advanced NSCLC. Corresponding PTVs demonstrated a respective, but smaller decrease. In the actually delivered plans (dPlan), both conformity and homogeneity indices were impaired compared to the initial plans (pPlan). This resulted in higher doses to the healthy lung tissue than planned. By means of weekly plan adaptation, healthy lung tissue could be spared and conformity and homogeneity indices reached levels of the initial plan.

In the current study, resolution and soft-tissue contrast of daily CB-CTs were sufficient to visualize lung tumor shrinkage. Michenzi et al. assessed the accuracy of CB-CT for quantifying NSCLC tumor volume changes and reported a high correlation between CB-CT and the gold standard contrast-enhanced diagnostic CT (42). In our study, continuous tumor shrinkage during radiotherapy was detected to an extent in line with other studies reporting residual volumes of 49–75% and volume decreases of 1.2–1.5% per fraction (15, 16, 43, 44). However, detection of treatment response and volume changes of mediastinal lymph node involvement was found to be challenging on CB-CT. Berkovic et al. also emphasized that CB-CTs lack contrast to truly distinguish between lymph node and other surrounding tissue, which only allows for adaptation on the lung-lymph node boundary (17). Similarly, in our study, primary tumors could easily be adjusted based on CB-CT, but lymph node tumor volumes could only be adapted to a limited extent. Notably, others have described lymph node shrinkage from 0 to 37% during radiochemotherapy for NSCLC (45–48). The soft-tissue limitations of CB-CT described above might be a reason why PTV shrinkage was less pronounced than GTV shrinkage in this work.

In the present study, weekly adaptation of target and OAR volumes enabled assessment of actually delivered doses. This allows for quality control which is not routinely performed in radiotherapy. Impaired conformity and homogeneity indices as well as higher actual MLD and lung V_20Gy_ than planned demonstrate that the actual treatment is inferior compared to the theoretical plan. A previous study also revealed higher lung doses (MLD, V_20Gy_, V_30Gy_) if adjusted OAR, i.e. actual volumes at a specific fraction, were considered (16). Others also observed differences between delivered and planned GTV and OAR doses, albeit not significant (15, 49). A radiotherapy plan at the moment of approval is an ideal, theoretical plan which does not perfectly depict dose variations due to volume changes and position inaccuracy over the course of treatment. This general problem of radiotherapy may be one possible explanation for local treatment failure despite guideline-specific dose prescription. IGRT and ART are possible means to overcome or at least reduce this problem.

Higher actual lung doses than planned resulted in a lung NTCP of 15.2% instead of 9.6%, a 58.9% increase. Assuming validity of the models established by Niemierko et al. which are based on the frequently cited paper by Emami et al (50)., an increase of that extent is clinically highly relevant. Numerous data on risk factors for development of radiation pneumonitis has been published, also summarized in reviews and meta-analyses (51–55). Dosimetric parameters associated with radiation pneumonitis include lung V_20Gy_ (52) and mean lung dose (56). Especially patients at older age, poorer performance status and with lung comorbidities have an elevated risk of developing radiation pneumonitis (52, 57). For other OAR than the lung (heart, esophagus, spinal cord), delivered doses and NTCP risks did not differ significantly from the initial plan.

### Potential of CB-CT-Guided ART for Optimizing PTV Coverage and Sparing of OAR

Using weekly CB-CT-based ART, doses to healthy lung tissue and resulting lung NTCP could be decreased to levels of the initial plan. Conformity and homogeneity could be restored to match the quality of the initial, theoretical plan. Prior studies also showed the potential of ART to reduce OAR doses (15, 17, 27, 58). In detail, Guckenberger et al. achieved reduced mean lung dose and dose to the spinal cord (15). Moller et al. showed a significant reduction of the mean lung dose (27). Berkovic et al. demonstrated that one single adaptation after 15 or 20 fractions may be sufficient to significantly reduce mean heart dose, mean esophagus dose, and spinal cord D2cc (17). In our study, some doses to OAR other than the lung (namely mean esophagus and mean heart dose) could be decreased by ART, but not significantly. Of all OAR, the healthy lung seems to benefit most from ART. This might be due to the volume change related to tumor shrinkage, while heart, esophagus, and spinal cord remain unaffected by tumor shrinkage. Benefits from tumor shrinkage were detected to be higher if shrinkage occurred in close proximity to the respective OAR. The esophagus is a very motile organ and challenging to contour on CB-CTs with poor soft-tissue contrast. Varying position and degrees of filling as well as contractions due to swallowing might impair reproducibility and thus dosimetric gains. The above discussed challenges in detecting lymph node regression close to the heart on CB-CT probably impaired the possibility to show significantly decreased heart doses for ART in the present study.

A potential risk of ART lies in the underdosage of microscopic tumor that was part of the initial GTV/CTV. As demonstrated in prior studies, this area is usually radiated at adequate doses despite ART (17, 20). Ramella et al. prospectively performed CT-based re-planning in 50 stage III NSCLC patients. After a mean follow-up of two years, overall local failure rate was 30%. Notably, marginal failure (i.e. inside of the initial PTV, but outside of the adapted PTV) occurred in only three patients (i.e. 6%), while in-field (i.e. covered by both initial and adapted PTV) failure was observed in ten patients (i.e. 20%) (26). As the reported local failure rate was in line with data from the RTOG 0617 trial (59), the authors concluded that adaptation did not increase the risk of local failure.

Regarding the optimal time for single adaptation, evidence is heterogeneous. Generally, the time of maximal shrinkage has to be weighed against the potential remaining benefit. In our study, GTV shrinkage was most pronounced in weeks 4 and 3. PTV shrinkage was pronounced in these weeks as well, but even higher in week 6. However, single adaptation in week 6 would not result in significant benefits, as the vast majority of the dose is already applied by then and only a few fractions remain. Berkovic et al. determined the optimal time point for single adaptation to be after 15 or 20 fractions, depending on chemotherapy sequence (16). In another study using 4D PET/CT scans in weeks 0, 2, 4 and 7 during RT, the optimal time for adaptation could not be determined solely based on image parameters (60). Given the data of Berkovic et al. and our study, the optimal time point for CB-CT-based single adaptation is after the third or fourth week of radiotherapy.

### Potential of ART-Based Dose Escalation

The aim of plan adaptation based on tumor regression may not only be the reduction of toxicity, but also the potential benefit of dose escalation. For NSCLC patients, a 1 Gy biological effective dose (BED) increase in radiotherapy dose intensity is associated with approximately 3 and 4% relative improvement in locoregional control and survival, respectively (61). The RTOG 0617 trial investigated dose escalation for patients with locally advanced NSCLC (74 Gy instead of 60 Gy), but reported significantly reduced survival in the high-dose arm (59). Several hypotheses for these results have been discussed; among them an increased mortality risk due to comparably high lung and heart doses (62–64). As OAR dosimetrics, despite weekly adaptation, did not provide promising potential for dose escalation at an isodose level of the OAR, the latter was not performed in our study. On the contrary, Guckenberger et al. reported about the possibility of GTV dose escalation from 66.8 ± 0.8 Gy to 73.6 ± 3.8 Gy with adaptation once or twice (at weeks 3 and/or 5) for patients with locally advanced NSCLC. However, only the mean lung dose was kept at an isodose level with this strategy and dose escalation was only afforded by mean maximum dose escalation of the spinal cord from 42.5 ± 5.0 Gy to 46.3 ± 5.3 Gy. Furthermore, in five of 13 patients, tolerance of the spinal cord (50 Gy) was exceeded in the study by Guckenberger et al (15)..

Gilham et al. analyzed the potential of plan adaptation based on PET/CT response after 50 out of 60 Gy for 10 patients with locally advanced NSCLC. Dose escalation could be achieved in four patients, while in the remaining six, normal tissue constraints would have been exceeded when applying an isodose approach for the lungs and the esophagus, but again not for the spinal cord (21).

Mathematically, a specific volume decrease (proportional to diameter to the power of 3) results in a smaller respective surface decrease (proportional to diameter to the power of 2). As IMRT fields are 2d projections, the dosimetric effect of a given GTV reduction can be smaller than expected. The main achievement of adaptation demonstrated in this work is the restoration of initial plan quality.

### Limitations

As limited soft-tissue contrast of CB-CT does not allow for adaptation of mediastinal lymph nodes (16), ART was restricted to the primary tumor in the current study. For adaptation of mediastinal CTVs/PTVs, contrast-enhanced 3D or 4D-CT would have been mandatory. Regarding the results for gEUD, NTCP, and TCP, it should be noted that plans were not primarily optimized according to these parameters. Thus, dosimetric benefits from adaptation would probably have been higher if these parameters had been used for primary plan optimization. Possible errors emerging from deformable image registration and dose accumulation should also be considered and remain an issue in all studies using CB-CT for dose calculation (32, 33, 65). The deformable image registration algorithm used in the present work has been validated before. With a target registration error of 1.17 ± 0.87 mm and a DICE similarity coefficient of 0.98 for both lungs, robust registration has been demonstrated (31). For CB-CT-based dose calculation in Raystation, published dose differences compared to diagnostic fan-beam CT range from below 1% (66) to 1.4% (34). Even though potential impact of CB-CT limitations on the results of this study remains possible, a large systematic error confounding the main findings seems unlikely given the reassuring results of previously published validation studies.

Plans were re-optimized using the same arc configurations as for the initial plan. Furthermore, this in silico study only comprised a small patient cohort and did not include clinical outcome parameters.

Significance of dosimetric benefits from ART might be more pronounced in larger studies. However, the process of weekly ART requires high input of time and workforce. Thus, reduced frequency of adaptation should be further evaluated, as present evidence is contradictive. Predictive atlases (67) or radiomic approaches (68) could be a solution to predict tumor shrinkage and necessity of adaptation based on initial images. Using deep learning approaches, target volume, and OAR delineation will probably be further automatized in the future (69).

Improved computer methods such as deformable registration of planning CTs to create virtual CTs matching up-to-date CB-CTs (19, 70) are also promising. Further advances in image reconstruction and procession, e.g. using deep learning (70), could enhance CB-CT quality and allow for precise delineation of lymph nodes. Advanced motion management strategies such as an internal target volume (ITV) or deep inspiration breath hold techniques were not used in this study. In our institution and most other big centers in Germany, these strategies are only used in pulmonary stereotactic body radiotherapy (SBRT). Implementation into clinical routine of normofractionated radiotherapy of NSCLC could help to further spare dose to healthy OAR. Surface guidance, which is increasingly used in clinical radiotherapy, can serve as a surrogate for breathing motion and also help to reduce OAR doses (71). Finally, MR-guided radiotherapy using MR-linacs, which is increasingly used for pulmonary SBRT, might also improve radiotherapy of locally advanced NSCLC by on-table real-time adaptation (72, 73).

### Conclusion

Weekly CB-CT-based adaptive radiotherapy is feasible in a clinical setting. The main benefit of ART is to maintain and assure radiotherapy quality at the initially planned level. CB-CT-based ART allows for sparing of healthy lung tissue and maintaining high plan conformity.

## Data Availability Statement

The datasets presented in this article are not readily available because data are only available in the treatment planning system. Requests to access the datasets should be directed to PD Dr. Juliane Hörner-Rieber, juliane.hoerner-rieber@med.uni-heidelberg.de.

## Ethics Statement

The studies involving human participants were reviewed and approved by Ethikkommission der Medizinischen Fakultät Heidelberg, Alte Glockengießerei 11/1, 69115 Heidelberg, Germany. Written informed consent for participation was not required for this study in accordance with the national legislation and the institutional requirements.

## Author Contributions

JH-R and JD developed the concept and drafted the study. PHo and CL analyzed the data. CL, PHa, and MS performed the planning calculations. SA and JH-R performed tumor delineations. AM, MB, CS-A, and TB helped with OAR contouring. RE, FW, LK, and JD critically revised the manuscript and helped with advise and input. PHo, CL, and JH-R wrote the manuscript. All authors contributed to the article and approved the submitted version.

## Conflict of Interest

The authors declare that the research was conducted in the absence of any commercial or financial relationships that could be construed as a potential conflict of interest.
